# COVID-19 Burden on HIV Patients Attending Antiretroviral Therapy in Addis Ababa, Ethiopia: A Multicenter Cross-Sectional Study

**DOI:** 10.21203/rs.3.rs-699963/v1

**Published:** 2021-07-27

**Authors:** Dagmawi Chilot, Yimtubezinash Woldeamanuel, Tsegahun Manyazewal

**Affiliations:** University of Gondar College of Medicine and Health Sciences; Addis Ababa University; Addis Ababa University

**Keywords:** Coronavirus disease 2019 (COVID-19), Severe acute respiratory syndrome coronavirus 2 (SARS-CoV-2), HIV, clinical care, treatment, antiretroviral therapy, Ethiopia

## Abstract

**Background:**

There has been promising progress towards screening, testing, and retaining HIV patients in care in Ethiopia. Concern exists that possible disruptions in HIV programs due to COVID-19 could result in more HIV-related mortality and new HIV infections. This study aimed to investigate the real-time burden of COVID-19 on HIV patients attending antiretroviral therapy.

**Methods:**

We conducted a facility-based, multicentre, cross-sectional study among HIV patients attending antiretroviral therapy in 10 healthcare facilities in Addis Ababa, Ethiopia, in the COVID-19 pandemic period. Data was collected using adapted, interviewer-based questionnaires, and entered into Epi Info version 7 and exported to SPSS version 26 for analysis.

**Result:**

A total of 212 patients with HIV were included. Participants who missed visits for refill were 58 (27.4%). When the effects of other independent variables on appointments/visits for refill were controlled, the following characteristics were found to be the most important pridictors of missed appointments (*P < 0.05*): age ≥ 55 [AOR = 6.73, 95% CI (1.495–30.310)], fear of COVID-19 [AOR = 24.93, 95% CI (2.798-222.279)], transport disruption [AOR = 4.90, 95% CI (1.031–23.174)], reduced income for traveling to health facility [AOR = 5.64, 95% CI (1.234–25.812)], and limited access to mask [AOR = 7.67, 95% CI (1.303–45.174)], sanitizer [AOR = 0.07, 95% CI (0.007–0.729)] and non-medical support [AOR = 2.32, 95% CI (1.547–12.596)]. The participants were well aware of the COVID-19 preventive measures. The most costly COVID-19 preventive measures that cause financial burden to the patients were costs for buying facemasks (63.7%), disinfectants (55.2) and sops for handwashing (22.2). Participants who missed follow-up diagnostic tests were 56 (26.4%). Variables which were found to be statistically significant include the following: age ≥ 55 [AOR = 0.22, 95% CI (0.076–0.621)], partial lockdown [AOR = 0.10, 95% CI (0.011–0.833)], limited access to health services [AOR = 0.15, 95% CI (0.045–0.475)], reduced income for traveling to health facility [AOR = 0.18, 95% CI (0.039–0.784)], and unable to get mask [AOR = 0.12, 95% CI (0.026–0.543)]. Participants who missed counseling services were 55 (25.9%). In multivariate logistic regression the following were statisticaly significant: age ≥ 55 [AOR = 0.21, 95% CI (0.078–0.570)], fear of COVID-19 [AOR = 0.11, 95% CI (0.013–0.912)], reduced income [AOR = 0.17, 95% CI (0.041–0.699)], unable to get face mask [AOR = 0.19, 95%CI (0.039–0.959)], and partial lockdown [AOR = 0.08, 95% CI (0.008–0.790)].

**Conclusions:**

COVID-19 had a significant burden on HIV patients to attend their routine clinical care and treatment, which may lead to treatment failure and drug resistance. The impact was on their appointments for medication refills and clinical and laboratory follow-ups. Targeted initiatives are needed to sustain HIV clinical care and treatment services and improve the wellbeing of people living with HIV.

## Background

Coronavirus disease 2019 (COVID-19) could be the most catastrophic pandemic in modern history. It has infected over 173, 674,509 people globally and resulted in more than 3,744,408 deaths as of 09 June 2021 [1]. Countries have been taking strong preventive measures to reduce and curve the transmission [2-4]. Many health care professionals shifted and health facilities were repurposed into targeted COVID-19 centers to manage patients [5-7]. Evidence showed these measures have led to restrictions of health facilities to the management of emergency medical conditions and chronic diseases care and treatment services [8, 9]. In Ethiopia, COVID-19 imposed a burden on physical infrastructure and exacerbated the preexisting weaknesses of health systems. As the country has limited numbers of hospitals and health centers, it presented a significant challenge to manage the pandemic and other diseases simultaneously [10-13].

By the end of 2020, it was estimated that 37.6 million people have Human Immunodeficiency Virus (HIV) infection globally and 1.5 million are newly infected. Only 27.4 million of them are on treatment with antiretroviral therapy (ART), which means 10.2 million (27%) people are still to come [14]. HIV remains highly prevalent in Africa, accounting for more than 67% of the people living with HIV/AIDS (PLWH) worldwide [15]. Concern exists that possible disruptions in HIV programs due to COVID-19 could result in more HIV-related mortality and new HIV infections.

The double burden of COVID-19 and HIV is one of the major health challenges especially in developing countries with high HIV prevalence [16-18]. PLWH might be particularly at high risk for infection with poor clinical outcomes [19-21]. Containment measures, disruptions to supply chains, and loss of income have the potential to exacerbate the impacts of the pandemic on HIV patients [22]. While these impacts will vary significantly across countries, some recommended providing ART for 3–6 months and others began to offer home delivery services through volunteers to reduce adverse health outcomes [23, 24]. the extensive demand for physicians has led to the rescheduling of routine HIV patients reviews and hospital visits [25-27]. Fear of COVID-19 exacerbated food insecurity and COVID-19 protective behaviors hindered voluntary HIV testing and healthcare services.

Many countries warned that they are at risk of stock-outs of antiretroviral (ARV) medicines and some have critically low stocks as a result of the pandemic [28]. In addition, PLWH were doubtful regarding the availability of ART services and regarding which HIV clinic to attend in the pandemic period [29].

There are limited real-time patient-level researches regarding how effective and useful country-level COVID-19 interventions were for HIV patients. As well, the impact of the COVID-19 pandemic on HIV at a population level is not well known. With the limited level of evidence in the world and as to our knowledge, no research was done regarding the impact of the pandemic on patients attending HIV care and treatment services in Ethiopia. There is an urgent need for adequately powered studies that investigate the impact of COVID-19 on HIV clinical care and treatment to augment the health of people living with HIV.

Thus, this study aimed to investigate the real-time burden of COVID-19 on people living with HIV who were attending ntiretroviral therapy facilities in Addis Ababa, Ethiopia.

## Methods

### Design and setting

A multi-center study was carried out at ten primary health care centres in Addis Ababa, from April 15 to March 30, 2021. The city has 10 sub-cities and 116 woredas and has different government health facilities including six hospitals and 106 public health centers. In Ethiopia, the COVID-19 pandemic is higher in the capital Addis Ababa [30]. Addis Ababa is the highest in HIV prevalence next to Gambella regional state [31]. The study was conducted in 10 health facilities, one in each sub-city which has high HIV patient flow.

### Participants

In this study, the source population was all HIV patients of age > 18 years attending care and treatment in the selected health centers. The study population were those who were attending care and treatment services during the data collection period. Participants were included if they were I) patients with HIV, as confirmed within the study facilities or result referred from another health facility; II) man or woman aged ≥ 18 years; III) volunteer to participate in the study. All eligible participants who have been attending clinical care and treatment in those study sites during the data collection period were considered using a predetermined sampling procedure (Table 1).

### Data collection

Adapted, pre-tested and structured questionnaire used to collect primary data for the assessment of the overall impact of COVID-19. The data collection instrument was developed in English and was translated to Amharic, and later back-translated to English to check for any inconsistencies or distortion in the meaning and concepts of the words by another person. Eligible participants who were attending the selected health centers were invited to participate. Participants were given information about the study through an information sheet and signed a consent form if they agreed to be part of the study. The data collectors and supervisors were trained before the actual data collection period regarding the approach, objective of the study, and ethical issues. The data collection was interviewer-administered and the questionnaire includes sections such as sociodemographic characteristics, awareness about preventive measures, care and treatment services.

### Data analysis and interpretation

All questionnaires were checked for completeness every day by the principal investigator and supervisors. Data cleaning was conducted at the end of the data entry. The analysis was done using bivariate and multivariate logistic regression to observe the effects of independent variables on the outcome variable while simultaneously controlling for other potential confounding factors. The raw data entered into Epi Info version 7 to control entry errors and exported to SPSS 26 for analysis.

## Results

### Socio-Demographic Characteristics

A total of 212 HIV patients were enrolled in the study, with a response rate of 100%, and 133 (62.7%) were female. Of the total, 103 (48.6%) were in the age group 35–54 years. Most of them (41.5%) were married and 59 (27.8%) had attended primary education. One hundred and forty-six (68.9%) were Orthodox Christian, and 24.1% were governmental employees (Table 2).

### Most effective preventive measure of COVID-19

Most participants (86.8%) responded “Cover mouth nose with facemask” is the most effective preventive measure of COVID-19. Study participants' responses on preventive measures such as “stay at home” and “use disinfectant” were 77.4%, 76.4% respectively (Table 3).

### The financial burden of COVID-19

The most costly COVID-19 preventive measures that cause financial burden to the patients were costs for buying facemasks [135 (63.7%)], disinfectants [117(55.2%)], sops for handwashing [47 (22.2%)] (Table 4).

### HIV care and treatment services during COVID-19

Participants who obliged to chage health centre were 3 (1.4%), and 27 (12.7 %) denied health services. Almost all participants said health care providers were polite and respectful (99.5%), willing to listen and answer their questions (99.5%), give attention to their individual needs (99.1%) (Table 5).

### Main barriers to access health care during the pandemic

**Figure 1:**
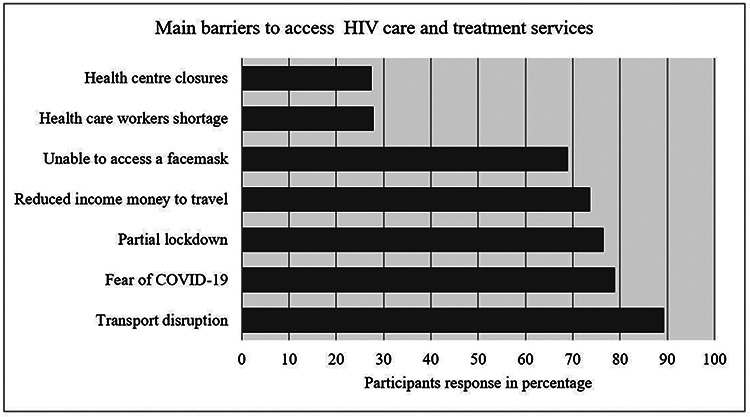
Response of study participants on barriers to access health care and treatment during the pandemic, Addis Ababa, Ethiopia May 2021.

Among study subjects, 189 (89.2%) said transport disruption was the main barrier to access health care. Fear of getting infected with COVID-19 (78.8%) was the second main barrier for the participants [figure 1].

### COVID-19 precaution measures in healthcare facilities

Among participants, 143 (67.5%) responded that health centers provide screening services for COVID-19 and all health professionals wear masks. Participants responded that there was water (97.2%) and soap (95.8%) at the gate of the healthcare facilities, but not sanitizer (74.1%) (Table 6).

### Medications and follow-ups during COVID-19

Among the total participants, 125 (59.0%) said ordered drugs are available. Two hundred (94.3%) were able to collect their multi-month drug supply. Participants who missed appointments, follow-up tests, and counseling services were 58 (27.4%), 56 (26.4%), and 55 (25.9%) respectively (Table 7).

### Logistic Regression analysis of missing appointments/visits for medication refill variable

By Bivariate and Multivariate Logistic Regression analysis of missing appointments/visits for medication refill variable, independent variables such as age, education, fear of COVID-19, transport disruption, reduced income, unable to access mask, sanitizer available, multi months drug supply, cost of disinfectant, non medical support since COVID-19 were significantly associated (Table 8).

### Logistic Regression analysis of follow-up tests variable

In Bivariate and Multivariate Logistic Regression analysis of follow-up tests variable, the following variables found to be significant: age, denied health services, reduced income/ money to travel, partial lockdown and unable to access face mask(Table 9).

### Logistic Regression analysis of counseling variable

Bivariate and Multivariate Logistic Regression analysis of counseling variable, factors such as age, education, fear of COVID-19, reduced income money to travel, unable to access face mask and partial lockdown were significant(Table 10).

## Discussions

To the best of our knowledge, this study was the first of its kind to assess the impact of COVID-19 on HIV care and treatment services in Ethiopia. We studied the overlap between two ongoing pandemics (HIV and COVID-19) in Ethiopia. The findings underscore several factors rendering HIV care and treatment services more difficult. A significant number of participants have missed appointments, follow-up tests, and counseling services due to COVID-19. COVID-19 containment measures taken by the government, patients’ sociodemographic characteristics, inconsistent access to personal protective equipment were the main factors that have hindered HIV patients' retention and adherence to their routine HIV care and treatment.

The patient living with HIV had great concerns about whether they are at high risk for the pandemic and the worse outcomes if they get infected with COVID-19. Research findings on these concerns have been in agreement with previous studies conducted elsewhere [18, 19, 33-36]. Studies indicated that though the pandemic affected the health care for all disease conditions, chronic patients such as people living with HIV are likely to be uniquely vulnerable [4, 37, 38]. In our findings, those who had formal education are more likely to have care and treatment services. This might be because respondents who had formal education may have a deeper understanding of the negative consequence if they missed their follow-up visits and they could have more tendency to request and access information about COVID-19 and its preventive measures.

Our results also indicated that HIV patients who had a fear of getting infected with COVID-19 and those who were elderly were more likely to miss appointments for care and treatment. This is also consistent with other findings [39-41]. It has been reported that the elderly and people with chronic conditions are more likely to be infected with COVID-19 and HIV patients may miss appointments as a result. COVID-19 containment measures taken in Ethiopia had a significant contribution in halting the spread of COVID-19 in Ethiopia; however, they had their own implications on HIV care and treatment services as the response from the HIV patients indicated. Transport disruption, partial lockdown that impaired mobility, and income reduction were significant factors for missing health care visits, which was in agreement with previous studies conducted in Ethiopia [10, 42,] and elsewhere in the world [43-50] that COVID-19 containment measure had a significant impact on patients’ access to healthcare facilities.

Undue expenses related to protective equipment including face masks sanitizers were a burden for the people living with HIV. The city of Addis Ababa introduced innovative measures providing ART medications for 3–6 months to mitigate these challenges. In our finding those who collect medications for 6 months were less likely to miss appointments for medication refill compared to those who took for 3 months.

Indirect impacts arising from the pandemic which reduced non-medical support had economical burdens. Indeed, health centers in Addis Ababa have had preeminent COVID-19 precaution procedures and measures to protect their clients from the pandemic. Availability of sanitizer, water, and soap at the health facilities’ gates encouraged the HIV patients to attend their routine care.

Our study has some limitations. The study was limited to healthcare facilities in Addis Ababa, and therefore may not be representative of Ethiopia. As the study design was a cross-sectional study, it does not show a causal relationship and only provides a view of the impacts of COVID-19 in a specific period. Otherwise, the study was based on real-time, patient-level primary data and it was conducted in a resource-constrained, high HIV burden country context.

## Conclusion

COVID-19 had a significant burden on HIV patients to attend their routine clinical care and treatment, which may lead to treatment failure and drug resistance. The impact was on their appointments for medication refills and clinical and laboratory follow-ups. Targeted initiatives are needed to sustain HIV clinical care and treatment services and improve the wellbeing of people living with HIV. Stakeholders such as the Addis Ababa health bureau, the ministry of health, and others should work in partnership to reduce the impact of this pandemic on those patients maintain their economic well-being.

## Figures and Tables

**Table 1:** Sampling procedure

Name of health facility	Sub-city	No of. HIV cases on ART
Addis raey HC	Addis ketema	20
Akaki HC	Akaki kality	50
Kebena HC	Arada	18
Goro HC	Bole	11
Addisu gebya HC	Gulele	16
Kazanchis HC	Kirkos	30
Alem bank	Kolfe	10
T/haymanot HC	Lideta	36
Woreda 02 HC	Nifas-silk lafto	10
Woreda 13 HC	Yeka	11

**Table 2:** Sociodemographic characteristics of respondents, Addis Ababa, Ethiopia, May 2021.

Variables Category		Frequency	Percentage
Sex	Male	79	37.3%
	Female	133	62.7%
Age	18–34	55	25.9%
	35–54	103	48.6%
	≥ 55	54	25.5%
Marital status	Single	50	23.6%
	Married	88	41.5%
	Widowed	40	18.9%
	Divorced	26	12.3%
	Separated	8	3.8%
Level of education	No education	46	21.7%
	Can read and write	30	14.2%
	Primary education	59	27.8%
	Secondary education	44	20.8%
	Diploma and above	33	15.6%
Religion	Orthodox	146	68.9%
	Muslim	36	17.0%
	Protestant	21	9.9%
	Catholic	3	1.4%
	Others	6	2.8%
Occupation	Student	3	1.4%
	Daily laborer	41	19.3%
	Merchant	22	10.4%
	Governmental employee	51	24.1%
	Private/NGO employee	43	20.3%
	Farmer	5	2.4%
	Housewife/unemployed	47	22.2%

**Table 3:** Respondents' awareness on COVID-19 preventive measure, Addis Ababa, Ethiopia. May 2021.

Variables Category		Frequency	Percentage
Stay at home	No	48	22.6%
	Yes	164	77.4%
Maintain physical distancing	No	87	41.0%
	Yes	125	59.0%
Avoid close contact	No	85	40.1%
	Yes	127	59.9%
Cover mouth nose with facemask	No	28	13.2%
	Yes	184	86.8%
Frequent handwashing with soap	No	43	20.3%
	Yes	169	79.7%
Avoid touching of eyes nose and mouth with unwashed hands	No	68	32.1%
	Yes	144	67.9%
Avoid mass gathering	No	92	43.4%
	Yes	120	56.6%
Restrict movement	No	98	46.2%
	Yes	114	53.8%
Use disinfectant	No	50	23.6%
	Yes	162	76.4%

**Table 4:** Respondents' financial burden of COVID-19 preventive measures, Addis Ababa, Ethiopia May 2021.

Variables Category		Frequency	Percentage
Facemask	No	77	36.3%
	Yes	135	63.7%
Soap for frequent hand washing	No	165	77.8%
	Yes	47	22.2%
Disinfectant	No	95	44.8%
	Yes	117	55.2%

**Table 5:** Response of study participants on health care facility and service delivery, Addis Ababa, Ethiopia May 2021.

Variables Category		Frequency	Percentage
Obliged to change the health centre because of this pandemic?	Yes	3	1.4%
	No	209	98.6%
Denied health services?	Yes	27	12.7%
	No	185	87.3%
Politeness & respect of health professionals?	Yes	211	99.5%
	No	1	0.5%
Willingness of professionals to listen and answer your questions?	Yes	211	99.5%
	No	1	0.5%
Attention of professionals to your individual needs?	Yes	210	99.1%
	No	2	0.9%
Staff seemed uncomfortable with you?	Yes	23	10.8%
	No	189	89.2%
Contact care provider when there is a health problem or comorbidities quickly?	Yes	101	47.6%
	No	111	52.4%

**Table 6:** Response of study participants on health facilities precautions for COVID-19 protection, Addis Ababa, Ethiopia, May 2021.

Variables Category		Frequency	Percentage
Health centre provide screening service for COVID-19?	Yes	143	67.5%
	No	69	32.5%
Health professionals wear the gloves during caregiving?	Yes	211	99.5%
	No	1	0.5%
Health professionals wear the mask during caregiving?	Yes	212	100%
	No	0	0.0%
Water available at the entrance of the health centre for hand washing?	Yes	206	97.2%
	No	6	2.8%
Soap available at the entrance of the health centre for hand washing?	Yes	203	95.8%
	No	9	4.2%
Sanitizer available at the entrance of the health centre for cleaning of hands?	Yes	55	25.9%
	No	157	74.1%

**Table 7:** Response of study participants on medications and follow-up, Addis Ababa, Ethiopia May, 2021.

Variables Category		Frequency	Percentage
Availability of ordered drugs?	Yes	125	59.0%
	Some	80	37.7%
	Not at all	7	3.3%
Non-medical support since COVID19?	Same as before	163	76.9%
	Slightly harder	15	7.1%
	Much harder	23	10.8%
	Impossible	11	5.2%
Have you had multi-month drug supply	Yes	200	94.3%
	No	12	5.7%
For how many months	3 months	90	42.5%
	6 monthes	110	51.9%
Have you missed appointments (visits)	Yes	58	27.4%
	No	154	72.6%
Follow-up tests done	Yes	156	73.6%
	No	56	26.4%
Counselling done on your medication or health status?	Yes	157	74.1%
	No	55	25.9%

**Table 8:** Bivariate and Multivariate Logistic Regression analysis of missing appointments/visits for medication refill variable, Addis Ababa, Ethiopia, 2021.

		Missed appointments		Odds ratio		
Variables Category		No	Yes	COR(CI)	AOR(CI)	P value
Age	18–34	43 (28.0%)	12 (20.7%)	1	1	0.252
	35–54	92 (59.7%)	11 (19.0%)	0.43 (0.175–1.048)	0.41 (0.091–1.875)	0.013*
	≥ 55	19 (12.3%)	35 (60.3%)	6.60 (2.823–15.434)	6.73 (1.495–30.310)	
Education	No education	14 (9.1%)	31 (53.5%)	1	1	0.001*
	Read + write	19 (12.3%)	11 (19.0%)	0.25 (0.096–0.670)	0.01 (0.001–0.165)	0.002*
	Primary edu.	50 (32.5%)	9 (15.5%)	0.08 (0.031–0.203)	0.02 (0.002–0.229)	0.052
	Secondary edu.	38 (24.7%)	6 (10.3%)	0.07 (0.024–0.201)	0.05 (0.003–1.022)	0.997
	≥ Diploma	33 (21.4%)	1 (1.7%)	0.01 (0.0012–1.021)	0.01 (0.001–1.002)	
Fear of COVID-19	No	44 (28.6%)	1 (1.7%)	1	1	0.004*
	Yes	110 (71.4%)	57 (98.3%)	22.80 (3.062-169.782)	24.93 (2.798-222.279)	
Transport disruption	No	22 (14.3%)	1 (1.7%)	1	1	0.038*
	Yes	132 (85.7%)	57 (98.3%)	9.50 (1.250-31.185)	4.90 (1.031–23.174)	
Reduced income	No	53 (34.4%)	3 (5.2%)	1	1	0.026*
	Yes	101 (65.6%)	55 (94.8%)	9.62 (2.873–32.219)	5.64 (1.234–25.812)	
Unable to access mask	No	64 (41.6%)	2 (3.4%)	1	1	0.024*
	Yes	90 (58.4%)	56 (96.6%)	19.91 (4.687–84.577)	7.67 (1.303–45.174)	
Sanitizer available	No	110 (71.4%)	47 (81.0%)	1	1	0.026*
	Yes	44 (28.6%)	11 (19.0%)	0.58 (0.278–1.231)	0.07 (0.007–0.729)	
For how many months	3 months	52 (35.9%)	38 (69.1%)	1	1	0.018*
	6 months	93 (64.1%)	17 (30.9%)	0.25 (0.129–0.486)	0.33 (0.132–0.825)	
Cost of disinfectant	No	85 (55.2%)	10 (17.2%)	1	1	0.023*
	Yes	69 (44.8%)	48 (82.8%)	5.91 (2.788–12.539)	16.64 (1.462-189.569)	
Non-medical support since COVID-19	Same as before	130 (84.4%)	33 (56.9%)	1	1	0.233
	Slightly harder	12 (7.8%)	3 (5.2%)	0.98 (0.263–3.693)	3.68 (0.434–31.204)	0.100
	Much harder	10 (6.5%)	13 (22.4%)	5.12 (2.064–12.705)	3.78 0.774–18.421)	0.044*
	Impossible	2 (1.3%)	9 (15.5%)	17.72 (3.655–85.987)	2.32 (1.547–12.596)	

* Statistically significant at p-value<0.05, COR = crude odds ratio at 95% confidence interval; AOR = adjusted odds ratio at 95% confidence interval.

**Table 9:** Bivariate and Multivariate Logistic Regression analysis of follow-up tests variable, Addis Ababa, Ethiopia, 2021.

		Follow up test		Odds ratio		
Variables Category		No	Yes	COR(CI)	AOR(CI)	P value
Age	18–34	12 (21.4%)	43 (27.6%)	1	1	0.073
	35–54	10 (17.9%)	93 (59.6%)	2.59 (1.041–6.472)	2.65 (0.913–7.670)	0.004*
	≥ 55	34 (60.7%)	20 (12.8%)	0.16 (0.070–0.382)	0.22 (0.076–0.621)	
Partial lockdown	No	1 (1.8%)	49 (31.4%)	1	1	0.034*
	Yes	55 (98.2%)	107 (68.6%)	0.04 (0.005–0.295)	0.10 (0.011–0.833)	
Denied health services	No	35 (62.5%)	150 (96.2%)	1	1	0.001*
	Yes	21 (37.5%)	6 (3.8%)	0.07 (0.025–0.177)	0.15 (0.045–0.475)	
Reduced income	No	3 (5.4%)	53 (34.0%)	1	1	0.023*
	Yes	53 (94.6%)	103 (66.0%)	0.11 (0.033–0.369)	0.18 (0.039–0.784)	
Unable to get mask	No	2 (3.6%)	64 (41.0%)	1	1	0.006*
	Yes	54 (96.4%)	92 (59.0%)	0.05 (0.013–0.226)	0.12 (0.026–0.543)	

* Statistically significant at p-value<0.05, COR = crude odds ratio at 95% confidence interval; AOR = adjusted odds ratio at 95% confidence interval.

**Table 10:** Bivariate and Multivariate Logistic Regression analysis of counseling variable, Addis Ababa, Ethiopia, 2021.

		Counsling done		Odds ratio		
Variable Category		No	Yes	COR(CI)	AOR(CI)	P value
Age	18–34	12 (21.8%)	43 (27.4%)	1	1	0.105
	35–54	10 (18.2%)	93 (59.2%)	2.59 (1.041–6.472)	2.28 (0.842–6.170)	0.002*
	≥ 55	33 (60.0%)	21 (13.4%)	0.18 (0.077–0.412)	0.21 (0.078–0.570)	
Education	No education	29 (52.7%)	16 (10.2%)	1	1	0.020*
	Read + write	11 (20.0%)	19 (12.1%)	3.24 (1.241–8.449)	3.68 (1.230–11.022)	0.000*
	Primary edu.	8 (14.5%)	51 (32.5%)	11.95 (4.572–31.251)	11.46 (3.906–33.615)	0.003*
	Secondary edu.	6 (10.9%)	38 (24.2%)	11.87 (4.142–34.047)	6.48 (1.921–21.876)	0.801
	≥Diploma	1 (1.8%)	33 (21.0%)	4.60 (0.391–15.227)	1.23 (0.238–6.412)	
Fear of COVID-19	No	1 (1.8%)	44 (28.0%)	1	1	0.041*
	Yes	54 (98.2%)	113 (72.0%)	0.05 (0.006–0.354)	0.11 (0.013–0.912)	
Reduced income	No	3 (5.5%)	53 (33.8%)	1	1	0.014*
	Yes	52 (94.5%)	104 (66.2%)	0.11 (0.034–0.380)	0.17 (0.041–0.699)	
Unable get face mask	No	2 (3.6%)	64 (40.8%)	1	1	0.044*
	Yes	53 (96.4%)	93 (59.2%)	0.05 (0.013–0.233)	0.19 (0.039–0.959)	
Partial lockdown	No	1 (1.8%)	49 (31.2%)	1	1	0.031*
	Yes	54 (98.2%)	108 (68.8%)	0.04 (0.005–0.304)	0.08 (0.008–0.790)	

* Statistically significant at p-value<0.05, COR = crude odds ratio at 95% confidence interval; AOR = adjusted odds ratio at 95% confidence interval.

## Abbreviations

AAU: Addis Ababa University
COVID-19: Coronavirus Disease 2019
SARS-CoV-2: Severe acute respiratory syndrome coronavirus 2
HIV: Human Immunodeficiency Virus
PLWH: people living with HIV
HC: Health center
ICU: Intensive care unit
PPE: Personal protective equipment
WHO: World health organization
AOR: Adjusted Odds Ratio
CI: Confidence Interval
